# Through the cleared aorta: three-dimensional characterization of mechanical behaviors of rat thoracic aorta under intraluminal pressurization using optical clearing method

**DOI:** 10.1038/s41598-022-12429-5

**Published:** 2022-05-23

**Authors:** Eijiro Maeda, Yoriko Ando, Kazuhiro Takeshita, Takeo Matsumoto

**Affiliations:** Biomechanics Laboratory, Department of Mechanical Systems Engineering, Graduate School of Engineering, Nagoya University, Furo-cho, Chikusa-ku, Nagoya, Aichi 464-8603 Japan

**Keywords:** Biomedical engineering, Imaging, Microscopy

## Abstract

The media of aortic wall is characterized by altering layers of elastin and smooth muscle cells (SMCs), along with collagen fibers in both layers, and plays a central role in functional and pathological remodeling such as hypertension and atherosclerosis. Because the arterial function is linked closely to the arterial wall internal structure, it is essential to investigate the alteration of the arterial microstructure during macroscopic deformation to understand cardiovascular pathologies. The present study adopted a tissue clearing method in three-dimensional mechanical characterization of rat thoracic aorta, and successfully observed changes in the structure of each of the three primary components of the aorta under intraluminal pressurization while maintaining tissue mechanical integrity and flexibility. Layers of elastic fibers and SMCs deformed greater on the intimal side than those on the adventitial side. Furthermore, there was a structural agreement in the alignment angle between SMC nuclei and elastic fibers on their intimal side, but not on the adventitial side. This is the first study that changes in the microstructure of three primary components of the aorta were visualized and evaluated through the aorta. The method established here would also be useful to understand tissue mechanics of other load-bearing soft tissues.

## Introduction

The aortic wall is characterized by three layers, the intima, media, and adventitia. Among them, the media mainly governs the mechanical behaviors of the aorta and plays a central role in the functional as well as pathological remodeling such as hypertension and atherosclerosis. The media mainly consists of elastin, collagen, and vascular smooth muscle cells (SMCs), each of which has elastic modulus at different orders. Namely, generally accepted values for the modulus of elastin, collagen, and SMC are reported to be approximately 0.6 MPa^1^, 1 GPa^1^, and 1–100 kPa^2^, respectively. Elastin and SMCs form distinct alternating layers, elastic lamellae (EL) and smooth muscle layer (SML)^3^ in the media, while collagen fibers are present in both layers in the media and the adventitia. The elastic lamellae are composed of circumferentially oriented elastin (71% of the total medial elastin), having small fenestrations within sheet-like EL structure^4–6^, and exhibit an undulated conformation in both the circumferential and axial direction in the unloaded condition^7–9^. This structural heterogeneity resulted in a nonlinear, viscoelastic mechanical behavior of the aorta under intraluminal pressurization.

Mechanical behavior of the aorta is generally described in two phases^10^: a large deformation in a low-pressure range and a small deformation in a high-pressure range. In the first phase in the low-pressure range, the aortic wall radially expands via circumferential stretching of elastic fibers and straightening of undulated collagen fibers, while in the second phase in the high-pressure range, which corresponds to within and above the physiological blood pressure, the aortic wall exhibits a limited expansion as stiff, straightened collagen fibers withstand mechanical stress^11^. During the tissue deformation, SMCs are also deformed mainly in the circumferential direction^12^, which could be a mechanical trigger of SMC functioning. Because the arterial function is linked closely to the arterial wall internal structure, it is essential to investigate the alteration of the arterial microstructure during macroscopic deformation in order to understand cardiovascular pathologies and consequent alterations of arterial functions and mechanics. Such information needs to be obtained from experiments conserving the unique three-dimensional arterial structure.

Recent attempts of three-dimensional characterization of aortic mechanical behaviors using intact aorta explants have demonstrated that a fraction of collagen fibers that remained undulated was higher in ELs than SMLs even in the high-pressure range^13^, suggesting that SMLs are more stretched than ELs during the pressurization. This may result in interlayer sliding during the circumferential deformation^14^. Meanwhile, this study also reported that strain levels in ELs and SMLs are at similar levels, and there were no statistically significant differences in strain levels from inner to outer layers of ELs as well as SMLs. These findings were obtained from mouse thoracic aorta, probably because of a limited transmittance of excitation two-photon laser through a thicker aorta specimen from larger animals^13,15^. Accordingly, there is a need to study the three-dimensional mechanical behavior of the aorta from different model animals under intraluminal pressurization to obtain a comprehensive understanding of vascular biomechanics, and to achieve this a suitable experimental set-up must be established.

Optical microscopy has been a powerful tool for characterizing mechanical behaviors of biological soft tissues including blood vessels. However, conventional confocal laser scanning microscope and even multiphoton microscope have a limitation in the depth that they can observe due to a high light scattering and absorbance of biological tissues (up to from several ten to two hundred micrometers), and thus three-dimensional observation of the deep inside of the tissue has been challenging. To overcome such difficulties, optical clearing methods have been proposed^16^. Although these techniques help to observe through a whole tissue explant or even a mouse as a whole^17^, this involved tissue fixation and removal of specific tissue components (e.g. lipids), resulting in solidified tissue samples, and thus, was not suitable for the observation of soft tissue deformation under mechanical loading^18,19^. Recently, a novel clearing method has been reported, which is capable of maintaining the viability of whole embryo, organoids, or even small model animals while it becomes transparent^20^. Indeed, this method successfully cleared tendon tissue in our previous study^21^. In the present study, we adopted this clearing method in three-dimensional mechanical characterization of rat thoracic aorta, and successfully observed changes in the structure of each of the three primary components of the aorta under intraluminal pressurization, while proving the maintenance of tissue mechanical integrity and flexibility. The method established here would be suitable for the three-dimensional mechanical characterization of not only blood vessels from large animals but also other types of load-bearing soft tissues.

## Results

### Structure observation of cleared aorta

**Figure 1:**
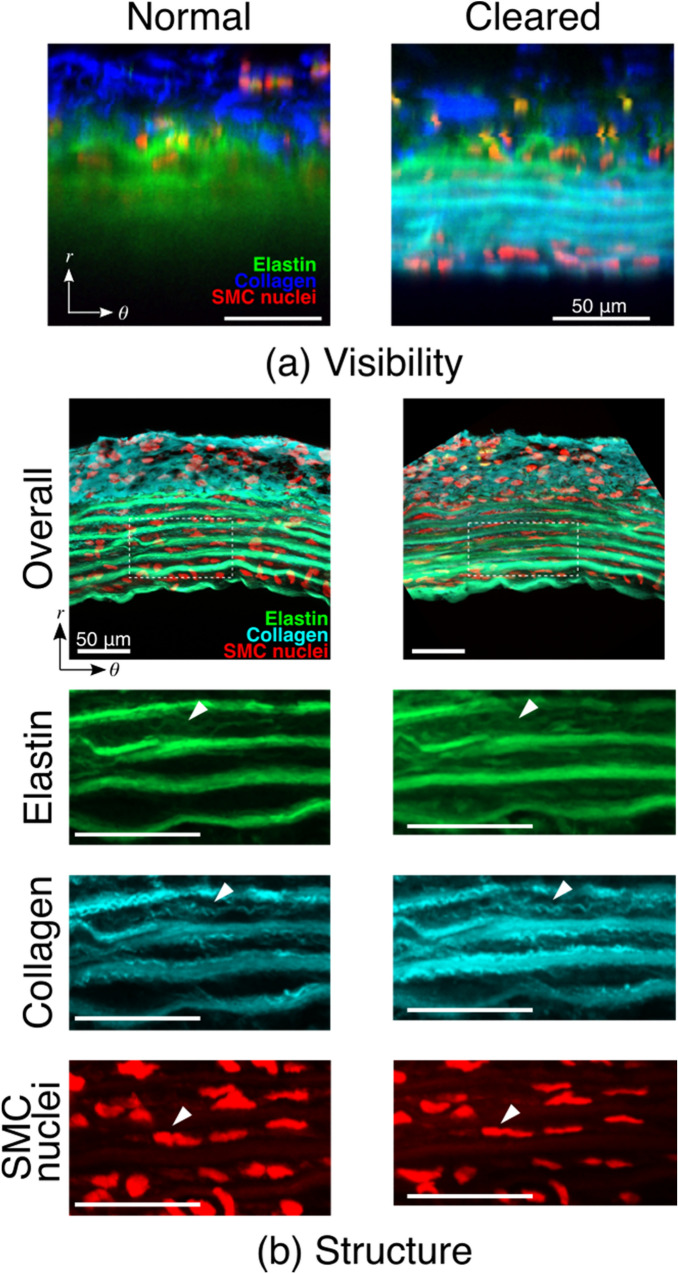
(**a**) The improvement of the visibility by tissue clearing method. A short segment of the rat thoracic aorta was isolated and cut open at the dorsal side, and the abdominal side was observed with a two-photon microscope from the adventitia toward the intima in normal, non-cleared state in PBS (left) and the same specimen in cleared state in the clearing solution (right). The images presented were the projection images in the radial-circumferential (*r *− *θ*) plane from the images originally obtained in the axial-circumferential plane. Elastin autofluorescence (green), collagen SHG (blue), and fluorescent signals from smooth muscle cell nuclei (red) were merged. Bars = 50 µm. (**b**) The preservation of microstructures of the rat thoracic aorta after the optical clearing. The cross-sectional images in the *r *− *θ* plane were obtained from normal, non-cleared aorta (left) and the same aorta following the clearing (right). Bars = 50 µm. Overall images (top) are the merged images of elastin autofluorescence (green), collagen SHG (cyan), and fluorescent signals from smooth muscle cell nuclei (red). Microstructures of each component are also presented individually, which are magnified images of a rectangular area indicated with white broken lines in each overall image. Arrowheads point to representative microstructure in each component observed both in the normal and cleared aorta. Bars = 50 µm.

First, we confirmed how the tissue clearing improves the visibility of the aorta specimen with a two-photon microscopy. In the aorta in a normal, non-cleared state in phosphate buffered saline (PBS), we were only capable of obtaining the images of the outmost EL, corresponding to the tissue depth of approximately 60 µm from the top of the adventitia surface (Fig. 1a). By contrast, following the optical clearing using 60% iodixanol solution (Optiprep, Abbott Diagnostics Technologies AS, USA) in PBS (clearing solution), we successfully observed through the aortic wall from the adventitia to the intima which existed approximately 90 µm depth from the adventitia surface (Fig. 1a). Note that weak fluorescent signals from SMC nuclei in the media was attributable to that these SMCs were probably still viable during the staining period.

We also examined if the present method of optical clearing made any structural alterations in the aortic wall. It was evident that microstructures of elastin fibers, collagen fibers, and smooth muscle cells were maintained well in the cleared aorta (Fig. 1b). It was also observed that the undulated elastic lamellae became relatively straightened and smooth muscle cell nuclei became narrow and elongated in the cleared state compared to the non-cleared state, possibly resulting in the increase of the outer diameter of the cleared aorta. The evidence coincided well with the previous findings of preserving biological organisms and cells with the same clearing method^20^ and proved that the current clearing technique made no remarkable structural changes.

### Pressure-diameter test

**Figure 2:**
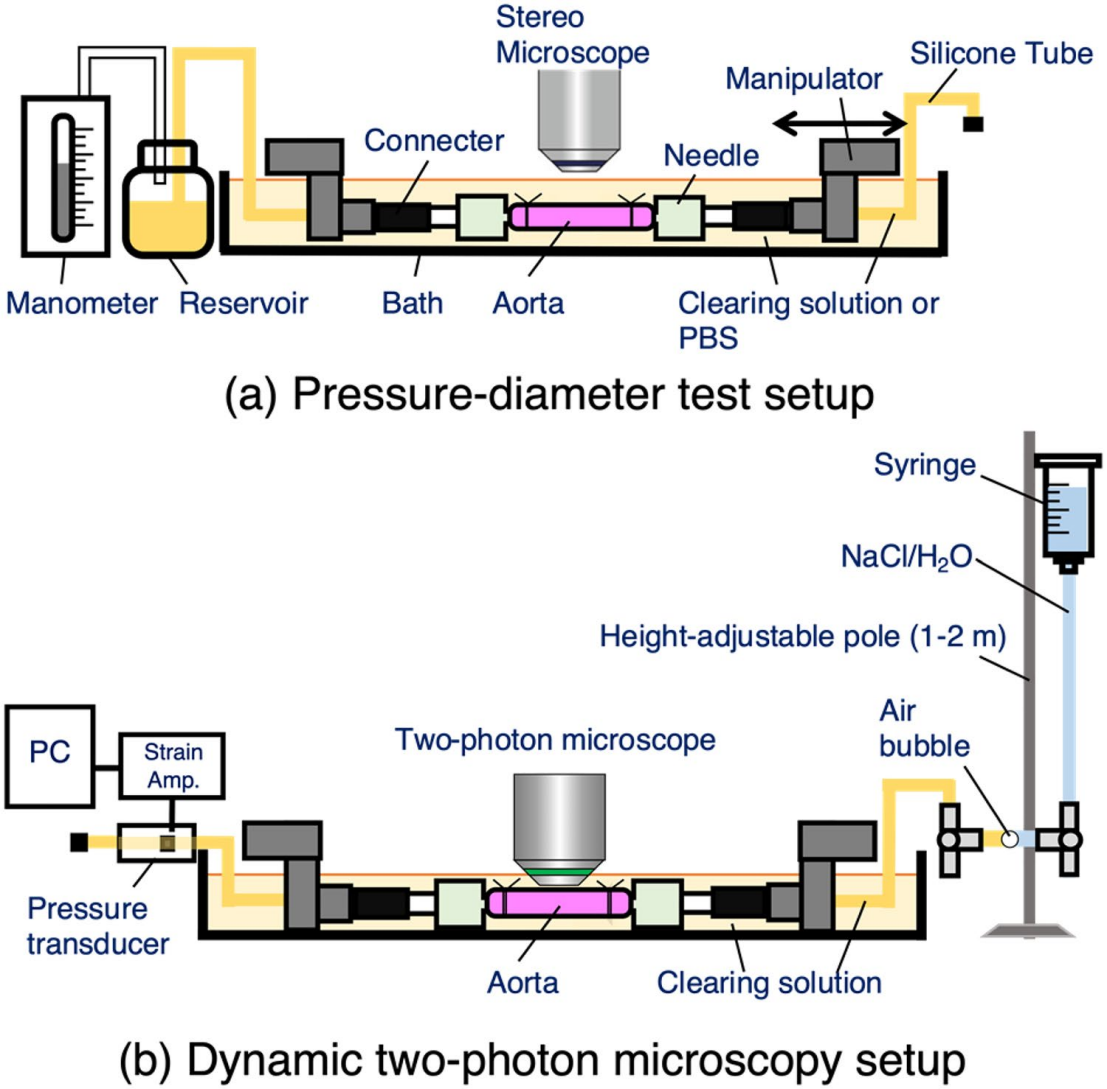
Schematic illustrations of the experimental setup for (**a**) pressure-diameter test and (**b**) dynamic two-photon microscopy.

**Figure 3:**
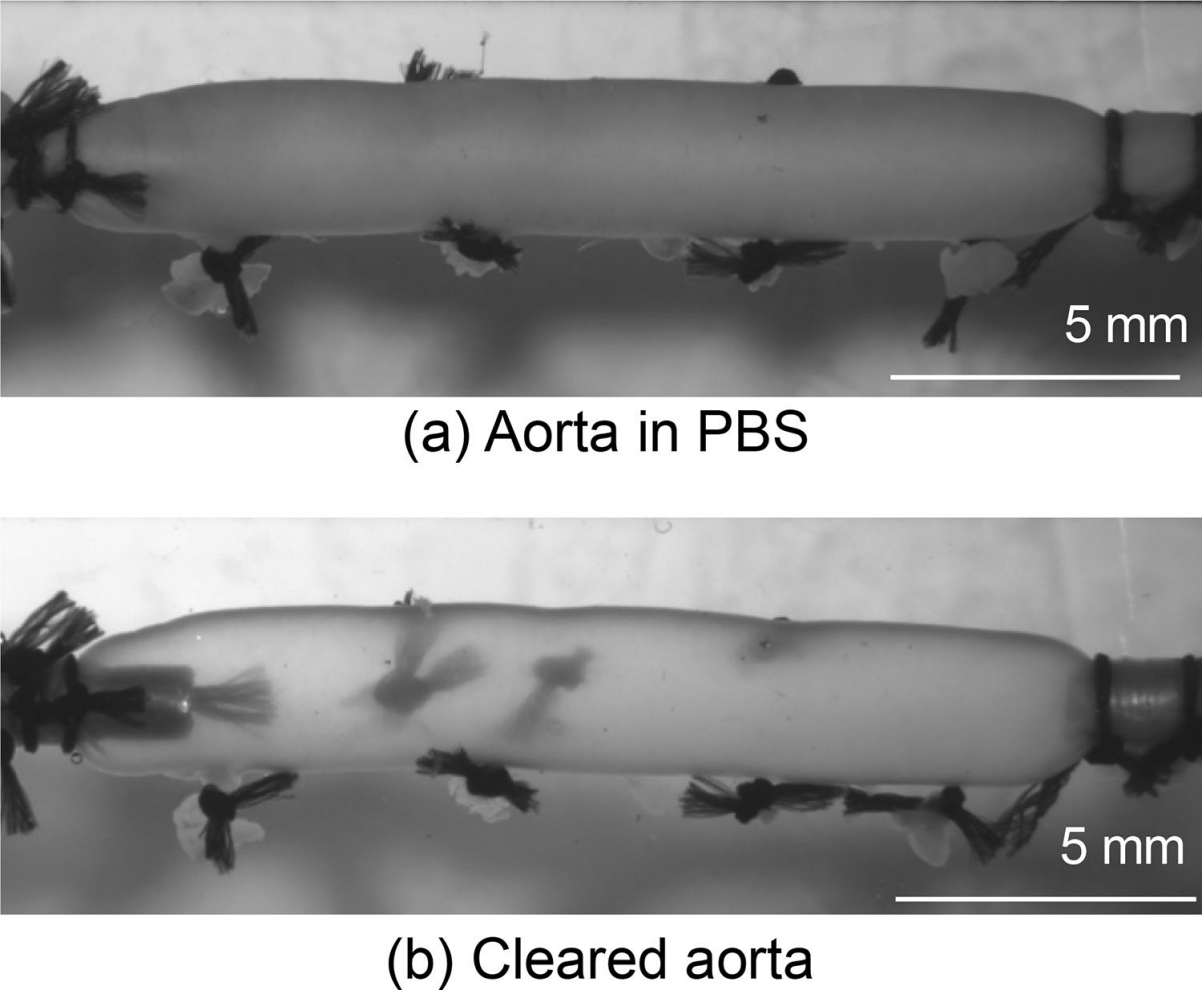
Representative photographs of rat thoracic aorta specimen subjected to the pressure-diameter test. (**a**) The specimen was tested in PBS, and (**b**) it was then cleared and tested in the clearing solution. Note that black knots, made for the closure of intercostal arteries, in the dorsal side (the backside) were clearly visible through the cleared aorta whereas they were invisible in the normal, non-cleared state.

**Figure 4:**
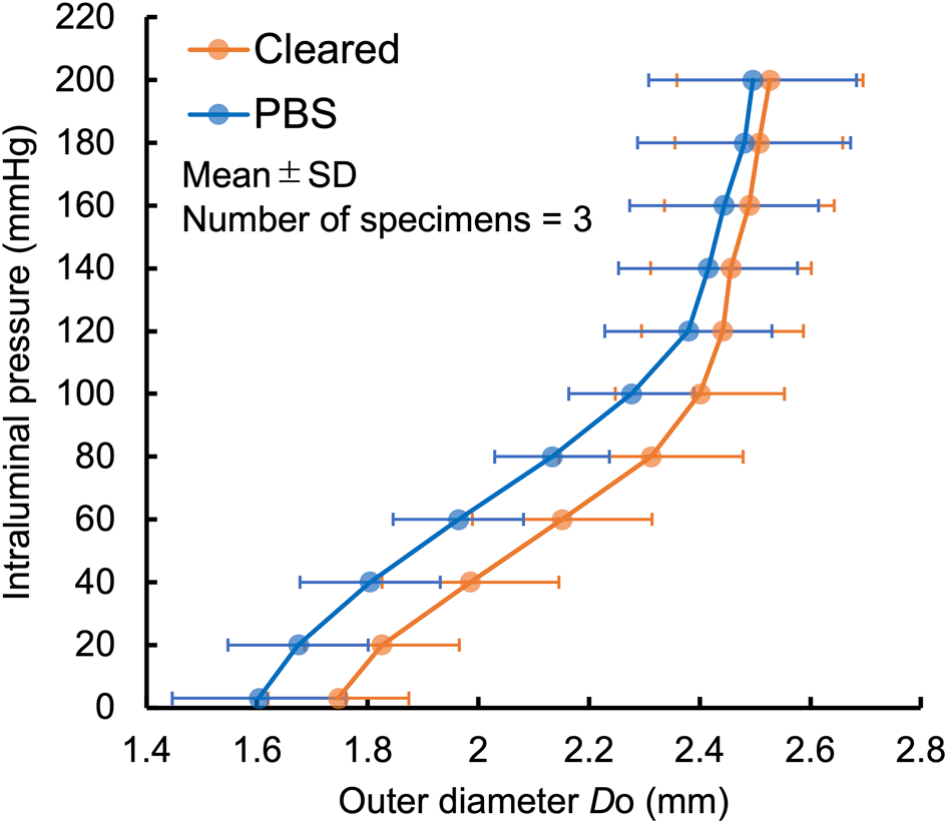
Pressure-diameter relationships of rat thoracic aorta tested in the normal state in PBS and in the cleared state in the clearing solution.

We next examined if the tissue clearing method alters the mechanical behavior of the aorta with a pressure-diameter test using a setup shown in Fig. 2a. In the clearing solution during the testing, the aorta turned to be transparent (Fig. 3), and this was a reversible change. The opacity of the cleared aorta was determined to be 0.18 ± 0.13 (mean ± SD, N = 3) (see Supplementary Fig. S1 for the analysis). There were no obvious differences in the deformation behavior between normal, non-cleared aorta specimens tested in PBS and cleared aorta specimens tested in the clearing solution (Fig. 4). Across the range of the intraluminal pressure examined, there was a general trend that the aorta diameter in the cleared state was relatively larger than that in the normal state. However, a nonlinear change in the diameter was consistent in both conditions; the aorta demonstrated low deformability at a pressure below 40 mmHg, a steady increase in diameter between 40 and 80 mmHg, and little change in diameter above 100 mmHg (Fig. 4). In two experiments, the specimen was placed back into PBS and stored at 4 °C after testing in the clearing solution, and the same testing protocol was repeated on the following day. The pressure-diameter relationships in both PBS and the clearing solution on day 2 were similar to those obtained on the first day (Supplementary Fig. S2), demonstrating the conservation of the tissue structure despite the tissue clearing process.

### Three-dimensional structure characterization

**Figure 5:**
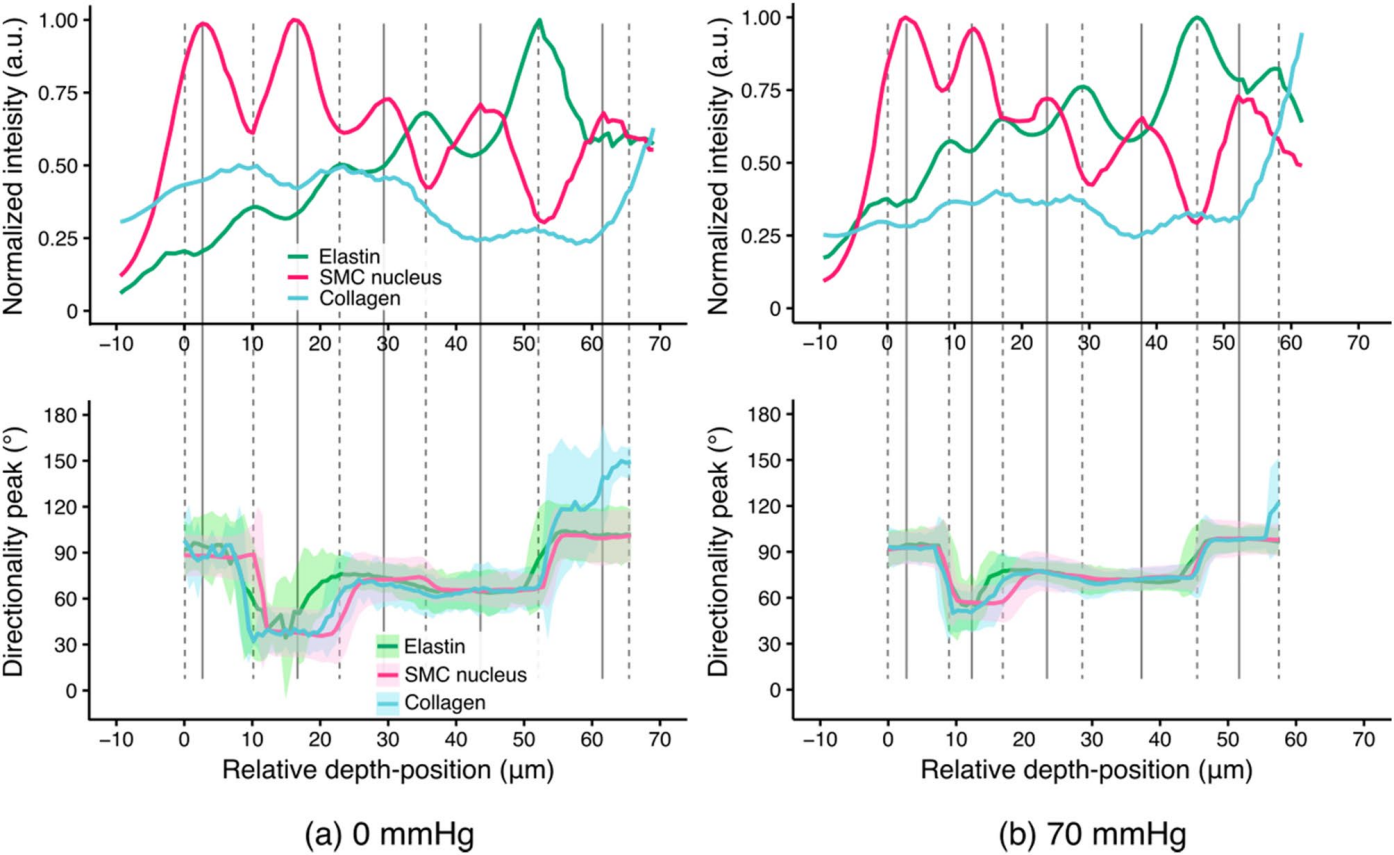
Representative signal intensity profiles of elastin autofluorescence, SMC nucleus staining, and collagen SHG (top), and the peak alignment angle of each component (bottom) obtained through the cleared aorta at an intraluminal pressure of (**a**) 0 mmHg and (**b**) 70 mmHg. Signal intensity was normalized to the maximum intensity of each respective component. The peak alignment angle (plotted with solid lines in the bottom) and the angle dispersion (equivalent to standard deviation, shown as bands) were determined using Directionality function in ImageJ/Fiji. The zero point of the horizontal axis corresponded to the peak in the first EL (EL1). Gray broken and solid lines drawn vertically indicate the depth-position of the local peaks of elastin autofluorescence and SMC nucleus fluorescence, respectively. The peak angle at 90° corresponds to the circumferential direction of the aorta.

**Figure 6:**
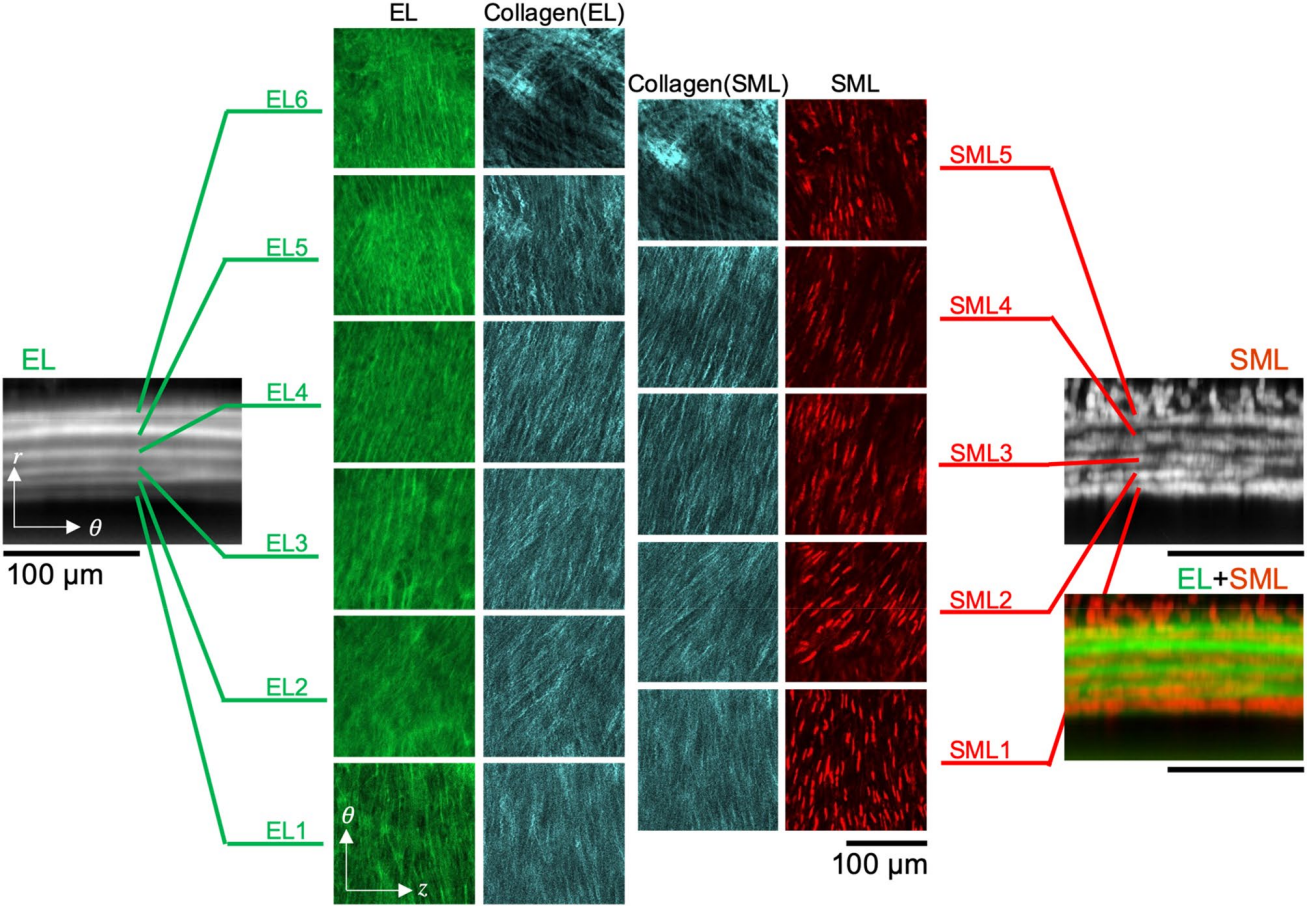
Layer-by-layer presentation of the microstructure of ELs, SMLs, and collagen fibers in both ELs and SMLs visualized in the cleared aorta. The axes of *r*, *θ*, and *z* indicate the radial, circumferential, and axial direction, respectively. Single slices from the depth-stack of EL, collagen, and SML on the *θ − z* plane are presented in the middle, exhibiting the microstructure of individual layers. The image of EL and SML on the *r *− *θ* plane (left and right, respectively) was created by projecting the image stack on *θ − z* plane to the *r *− *θ* plane (sum slices for EL and standard deviation for SML, respectively).

To observe the deformation of tissue structure through the aortic wall under an application of intraluminal pressure, an aorta specimen was cleared and subjected to dynamic multiphoton microscopy using an apparatus presented in Fig. 2b. Three-dimensional distribution of elastic fibers and SMC nuclei in the cleared aortas were clearly obtained using multiphoton microscopy as demonstrated in the signal intensity profiles (Figs. 5, 6). Collagen fibers were also visible across the thickness of the aortic wall despite a weaker signal intensity compared to elastin and smooth muscle cell nuclei. Figure 6 shows the microstructure of elastic fibers, the distribution of SMC nuclei, and collagen fibers at each of ELs and SMLs. The peak and the variability of the alignment angle distribution of each component throughout the depth-series images were also plotted in Fig. 5. Elastic fibers, SMC nuclei, and collagen fibers all followed a similar angle distribution profile; the majority of these components were aligned to 90° (the circumferential direction), with some layers aligned slightly offset from the circumferential direction (approximately 30°). When intraluminal pressure was applied, the amount of the dispersion of the orientation angle distribution became smaller. Indeed, in both ELs and SMLs, the peak angle of the overall alignment was consistently around 90° from 0 to 130 mmHg, with the variability decreasing with the increase in the pressure level (Supplementary Fig. S3). The dispersion was also reduced with the increase in the pressure in both ELs and SMLs (Supplementary Fig. S3).

**Figure 7:**
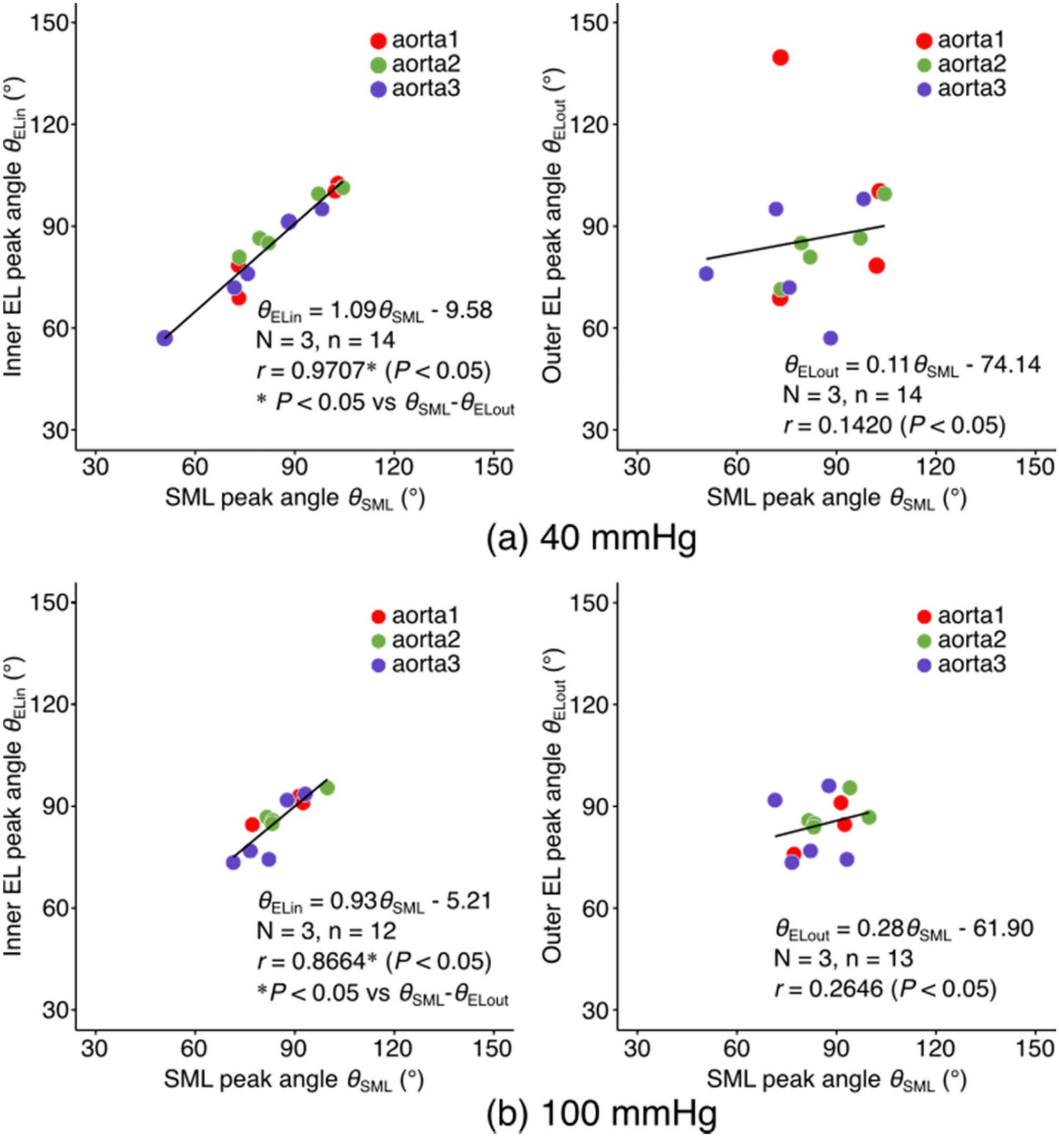
Correlation analysis of EL peak angles and SML peak angles. The correlation was calculated between SML and its inner EL (left) and between SML and its outer EL (right). The angles were obtained at the pressure level of (**a**) 40 mmHg and (**b**) 100 mmHg, respectively. The peak angle at 90° corresponds to the circumferential direction of the aorta. N, number of specimens; n, the total number of the pairs of SML and EL layers analyzed. Statistical analysis was performed for n.

We also analyzed whether the alignment of smooth muscle cell nuclei agreed with the alignment of elastic fibers in the inner (intimal side) or in the outer (adventitial side) elastin layer (Fig. 7). At the intraluminal pressure of 40 mmHg, SMC nuclei alignment was almost identical to the alignment of elastic fibers in the inner layer, but it did not agree with the elastin alignment in the outer layer (Fig. 7a). Indeed, the coefficient of the regression was significantly higher in the relationship between SML and the inner EL than that between SML and the outer EL (*P* < 0.001). At 100 mmHg, the trend was similar to that observed at 40 mmHg (Fig. 7b). The range of peak angles became smaller both in elastic fibers and SMC nuclei compared to the relationships at 40 mmHg. SMC nuclei alignment agreed well with the alignment of elastic fibers in the inner EL but poorly correlated with the alignment of elastic fibers in the outer EL. It was also confirmed that the coefficient of the regression was significantly higher in the relationship between SML and the inner EL than that between SML and the outer EL (*P* = 0.020).

### Strain analysis

**Figure 8:**
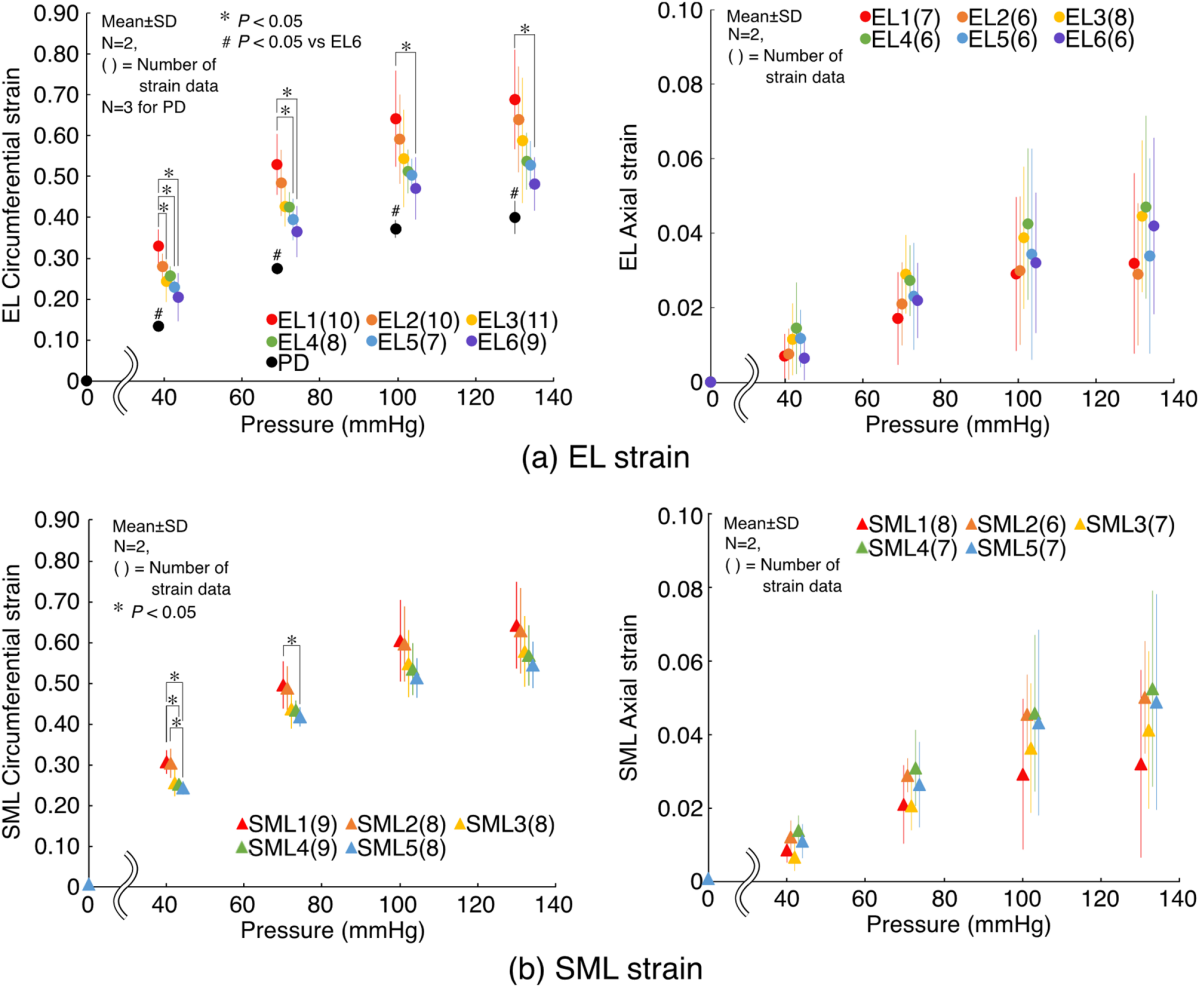
Circumferential (left) and axial (right) strain during pressurization in each layer of (**a**) ELs and (**b**) SMLs obtained from dynamic two-photon microscopy. PD in the plot of EL circumferential strain indicates the circumferential strain simply calculated from the changes in the apparent diameter during the pressure-diameter test results. N, number of specimens. Statistical analysis was performed for strain data indicated in the round brackets.

When the cleared aorta was pressurized, there was a clear trend that elastic fibers and SML in the inner layers deformed larger in the circumferential direction than the outer layers (Fig. 8). The level of the circumferential strain of the innermost EL (EL1) was significantly larger than the outermost EL (EL6) at all the pressure levels examined (*P* = 0.0006 at 40 mmHg, 0.0009 at 70 mmHg, 0.01 at 100 mmHg, and 0.01 at 130 mmHg). EL1 strain level was also significantly larger than EL 3 at 40 mmHg (*P* = 0.01) and EL5 at 40 and 70 mmHg (*P* = 0.004 at 40 mmHg and 0.01 at 70 mmHg), respectively. The innermost SML (SML1) exhibited a significantly larger circumferential strain level than SML4 at 40 mmHg (*P* = 0.039) and SML5 at 40 and 70 mmHg (*P* = 0.007 at 40 mmHg and 0.044 at 70 mmHg), respectively. The level of SML2 was also significantly larger than SML5 at 40 mmHg (*P* = 0.023). The strain levels of the inner SMLs were also larger than those of the outer SMLs at higher pressure levels, although the differences between layers were not statistically significant.

In contrast, there was no such clear trend in the strain levels in ELs and SMLs in the axial direction. In both ELs and SMLs, although the strain levels in the outer layers were slightly higher than the inner layers, the magnitudes of the axial strain were markedly lower than that of the circumferential strain. There were no statistically significant differences between layers at any of the pressure levels examined.

Collagen layer (CL) strain was analyzed at all pressure levels in one specimen as this was the only sample that collagen SHG images were clearly obtained across the thickness of the aortic wall. The strain was analyzed in collagen fibers in EL (CL(EL)) and those in SML (CL(SML)) separately, using the same method with the EL strain analysis. There was a general trend that the circumferential strain in the inner layers was greater than that in the outer layers (Supplementary Fig. S4), agreeing with the trends observed in the strain in ELs and SMLs.

## Discussion

In the present study, rat thoracic aorta was optically cleared through its depth while maintaining its mechanical compliance, and three-dimensional tissue behavior under intraluminal pressurization was characterized. In particular, mechanical strain in each layer of three aortic components, namely, elastin, smooth muscle cell, and collagen, was determined individually, revealing a significantly larger deformation in elastic lamella in the intimal side compared to the adventitial side. As far as the authors know, this is the first study to evaluate the changes in the microstructure of three primary components of the aorta in response to pressurization while maintaining its cylindrical structure.

In literature, there are several reports of the observation through arterial wall without optical clearing using a mouse model^22,23^. However, in case of rat thoracic aorta, it was difficult to look through the wall without optical clearing (Fig. 1a), possibly due to a thicker wall in rat. In the present study, the clearing method enabled us to observe fine structures of fibers and cell nuclei through the aorta by solving refractive index mismatches and calculate strain from the images. Therefore, the optical clearing makes it possible to observe through the wall of thicker arteries and to obtain microscopic images with fine details. The current technique provides the opportunity to study details of mechanical behaviors of blood vessels larger than mouse thoracic aorta.

The diameter of the aorta was slightly increased when it was incubated in the clearing solution (Fig. 4), and this might be due to an effect of tissue dehydration by the clearing solution. However, we did not confirm statistically significant change in the diameter of the aorta incubated in hypertonic saline solutions, even in a solution with the osmolality close to the clearing solution, over a period of 6 h (Supplementary Fig. S5). Accordingly, this small increase in the diameter may not be solely due to tissue dehydration. However, it was evident that ELs were slightly straightened, and SMC nuclei became narrow and elongated in the cleared aorta compared to non-cleared aorta (Fig. 1b). Therefore, physicochemical properties of iodixanol, the solute of the clearing solution, may influence the structure of the aorta and slightly inflate the aorta, which, however, possesses no significant impact on our experimental results and conclusions of the present study.

One of the important findings of the present study is that the tissue deformability of the cleared aorta was similar to that of noncleared original one; aorta in the both states exhibited nonlinear pressure-diameter relationship (Fig. 4). Such mechanical behavior under intraluminal pressurization was also reported in a previous study using the same artery from the same animal model^24^ as well as in the same and other arteries in other species^25,26^. The coincidence with other studies validates not only the mechanical data obtained in the present study but also the use of the current optical clearing method for dynamic two-photon microscopy experiment.

Characterization of aortic behavior under intraluminal pressure has been attempted in previous studies using two-photon microscopy^13,14,22,23,27^ and X-ray synchrotron^28^. However, findings obtained were limited to mechanical behavior of selected tissue components: changes in the orientation angle of adventitial collagen fibers under pressure ^27^, changes in the orientation angle of medial collagen fibers in ELs and SMLs as well as the differences in the degree of straightening of collagen fibers between EL and SML^13^, shear straining between EL and SML^14^, and unfolding of undulated elastic lamellae under pressure^28^. The present study stands apart from these studies in that the alignment and deformation of all three aortic components were analyzed from the innermost to the outermost elastic lamella. Nonetheless, it should be noted that the intensity of collagen SHG signal was varied between samples, especially that from collagen fibers in ELs and SMLs on the intimal side (EL1-3 in the present study). The possible reason for this was the absorption of SHG signals by collagen fibers in the adventitia. We kept the adventitia in place as this was necessary to preserve the original tissue structure of the aorta to replicate in vivo tissue mechanical behavior in the in vitro experimental setting. It was, however, highly likely that the adventitial collagen fibers absorbed collagen SHG signals and prevented the signals from reaching to the detector. As elastin autofluorescence and the fluorescence from cell nuclei were successfully observed even deep inside of the aortic wall, the two-photon excitation laser beam was not absorbed or scattered by the adventitial collagen fibers.

It was clearly exhibited that layers in the intimal side were deformed greater than those on the adventitial side (Fig. 8). In a previous study evaluating aortic wall remodeling to hypertension in rats, SMCs in the intimal side became hypertrophic to a larger extent compared to those in the adventitial side^29^. Our results that ELs on the intimal side (particularly EL1) were strained remarkably larger in a circumferential direction than those on the adventitial side (particularly EL6), and a similar trend observed in SMLs are well relevant to the previous findings. The present evidence supports the current notion that the hypertension in the aorta applies a large amount of mechanical strain (in the circumferential direction) to SMCs on the intimal side, and the cells respond to the increased strain (and associated stress) by structurally adapting to maintain the circumferential stress at a physiological level.

Besides the inter-layer differences in the strain magnitude, the inter-component differences in the strain magnitude as well as alterations in the alignment angles were assessed. However, we did not find statistically significant differences in the strain magnitudes among three components in each layer (Supplementary Fig. S6), or significant changes in the alignment angle during the inflation (Supplementary Fig. S7). Furthermore, a close investigation of the interactions between components was attempted, but it was difficult to perform at its current image resolution. It is hypothesized that we could visualize the mechanisms of how SMCs are stimulated in situ, as the frictional shear stimulations between their cell bodies and adjacent collagen fibers may occur due to different magnitudes of local strain between collagen fibers and SMCs. Such details of the SMC mechanical environment will be a target of our future work.

It was apparent in our strain data (Fig. 8) that the circumferential strain calculated from the diameter change in the pressure-diameter test (PD) was smaller than that calculated from dynamic two-photon microscopy (EL1–EL6). This follows a theoretical intramural distribution of circumferential strain along the radial direction in a thick-walled cylinder subjected to internal pressure. In other words, because the diameter of the aorta was measured from the images of the exterior of the aorta (Fig. 3), the measurement of strain was done more on the outside than EL6, resulting in a smaller strain value than EL6. Furthermore, it should be highlighted that local EL strain was measured in the abdominal side of the aorta. In previous studies of ours^30^ and others^31^, circumferential stretch of the aorta in response to intraluminal pressurization was larger in the abdominal side than in the dorsal side. Given that overall circumferential stretch of the aorta, which is equivalent to PD strain in the present study, can be estimated as an average of the ventral and dorsal strains, the abdominal circumferential strain was approximately 5% larger than the overall circumferential strain^30,31^. In the present study, EL6 circumferential strain was 7–10% larger than PD circumferential strain, which essentially agreed with these previous findings. Because EL6 strain was measured inside the aorta whereas PD strain was measured from the radially outmost surface of the aorta, EL6 strain could be larger than circumferential strain determined in the outmost surface on the abdominal side of the aorta. This difference in the radial position in strain measurement possibly led to our results that the difference between EL6 and PD strains was relatively larger than the reported differences between the ventral and overall circumferential stretch ratios. There were also some factors influencing the difference between PD and EL6, such as the difference in strain measurement method (measured from fiber structure in EL6 whereas measured from an overall artery size) and imaging method (two-photon microscope vs stereomicroscope).

Another important finding in the present study was the agreement in the alignment angle of the nucleus of SMCs and elastic fibers underneath them. Although SMCs are reported to anchor to ELs at both of their inner and outer sides^4,32^, the preference of the nucleus alignment to one side of ELs could be relevant to the structure development process of the aortic wall. The aortic structure is thought to be formed by the formation of a tubular structure by endothelial cells, followed by wrapping around by mesenchymal cells^33^. As the cells differentiate to SMCs, elastin is synthesized and deposited between layers of the cells^33^. Because the (inner) intimal side is deformed larger than the (outer) adventitial side with the intraluminal pressure, the cells may prefer to build a structure in their inner side, reinforced with fibers aligned to the direction of the deformation to withstand mechanical strain. Other mechanisms, such as a spontaneous formation of the aligned structure of elastic fibers could be possible, as a three-dimensional laminar structure, with highly organized collagen fibers in each layer, can be formed only with collagen molecules themselves via molecular crowding^34^. However, in the development of the aorta, this might not be the case as mesenchymal cells are accumulated prior to their elastin synthesis, and thus there might be no time and/or space for the spontaneous alignment formation of elastic fibers.

In the dynamic two-photon microscopy, the cell nucleus was fluorescently labeled with ethidium homodimer-III, which labels dead cell nuclei. This dye was selected based on the separation of the emission light wavelength from the specimen as well as the stainability. However, the good stainability with the dead cell marker indicates that SMCs within the cleared aorta were not viable. It has been well known that the SMC state between contracted and relaxed significantly affects mechanical behaviors of muscular arteries, but only a little alteration was observed in the behaviors of elastic arteries, such as carotid artery^35^. The rat aorta, used in the present study, is also categorized as an elastic artery. Therefore, although SMCs are not viable in the cleared aorta, mechanical behaviors of the cleared aorta are deemed to closely represent the behaviors of the normal, non-cleared aorta.

It has been reported that a tissue clearing technique influences tissue mechanics^36^. In this report, PBS supplemented with 80% propylene glycol (PG) was used for optical clearing of porcine aorta so that the tissue was dehydrated to obtain optical transparency. This technique also increased the stiffness of the aorta. Because the tissue clearing with PG was achieved via removal of light-scattering water from tissue and destabilization of high-order collagen structure by chemical agents in the clearing solution (i.e. PG)^37^, alterations in mechanical behavior were unavoidable. In the present study, on the other hand, we used 60% iodixanol solution (Optiprep) in PBS as the clearing solution, which has the estimated osmolality of 470 mOsm/L. This was remarkably lower than the estimated osmolality of 80% PG in PBS (11,000 mOsm/L)^38^. The supplementation of Optiprep into medium is aimed to reduce a mismatch of refractive indices between tissues/cells under imaging and the medium for the imaging^20^, but not to induce tissue dehydration to obtain tissue transparency. Indeed, it was demonstrated that porcine aorta remained opaque when it was incubated in 30% PG solution^36^ (estimated to be 4000 mOsm/L^38^). Therefore, the osmolality-induced dehydration by our clearing solution is supposed to be very small and not enough to make the rat aorta clear. It was also reflected in our results that mechanical behaviors of cleared rat aorta were similar to those of normal aorta (Fig. 4), possibly suggesting a small effect of the current clearing method on tissue mechanics. Tissue dehydration strategy may be appropriate to optically clear thick specimens like porcine aorta, and thus, it will be an interesting research topic in future study to compare tissue clearing methods for large aorta specimens.

One of the limitations in the present study is that we did not examine if the tissue clearing affect mechanical responses of the aorta, particularly at a microstructure level. Although we confirmed that the tissue clearing made no marked alterations in the structure of the aorta in a non-loaded state (Fig. 1) and in macroscopic mechanical behaviors (Fig. 4), these findings provide no confirmation that microstructural responses are also the same between normal and the cleared aorta. Therefore, future study should be conducted to address this issue.

In conclusion, we have established an experimental model of the characterization of aortic mechanical behavior through the thickness of the tissue by optical clearing, while maintaining tissue compliance. It was confirmed that the magnitude of the circumferential strain of elastic laminae was strongly dependent on the location within the aortic wall, and the orientation of smooth muscle cells is well agreed with that of elastic fibers located on their intimal side.

## Methods

### Isolation of thoracic aorta specimen

A total of 8 male Wistar rats (8–9 weeks old) were used in the present study. All animal experiments were approved by the Institutional Review Board for Animal Care at the Graduate School of Engineering, Nagoya University (approval number 18-8) and were performed in accordance with the Guide for Animal Experimentation, Nagoya University as well as the ARRIVE guidelines^39^. One animal was purchased at a time several days before the experiment and kept in an individual cage until sacrifice. All rats were maintained on a regular diurnal lighting cycle (12:12 light:dark) with ad libitum access to food (CLEA Rodent Diet CE-2) and water. Auto flushing system was used to keep the cage clean. The animals were housed in a specially-designated animal room ventilated and maintained at 25 °C throughout the year, and were allowed normal daily activities. No analgesia was provided to the rats before the sacrifice. Animal behaviors were carefully checked every day and no obvious abnormalities were evident in all animals before the use in the experiments. The experiments were designed to keep the minimum use of the animals.

The animals were sacrificed with carbon dioxide gas, and the tubular section of the thoracic aorta, with an in vivo length of approximately 40 mm, was obtained from each rat immediately after the sacrifice. The in vivo length between the proximal and distal cut positions was recorded in order to reproduce the physiological length in the in vitro experiments described below (an averaged stretch ratio of 1.4). The isolated aorta was placed in PBS while loosely connected soft tissues surrounding the aortic surface, including fat, were carefully removed while keeping the adventitia in place.

### Structure observation of cleared aorta

A short longitudinal section was taken from an isolated aorta and cut open on the dorsal side. The specimen was incubated in 5 µM ethidium homodimer-III (Biotium, USA) in PBS overnight at 4 °C to stain the nuclei of smooth muscle cells and was imaged under a two-photon microscope (A1R MP, Nikon, Japan) in a noncleared, normal state within PBS. The rectangular specimen was placed flat with the adventitia of the abdominal side facing upwards. Images on the axial-circumferential (*z *− *θ*) plane were obtained from the adventitia toward the intima with a 25 × water immersion objective lens and an excitation laser at a wavelength of 860 nm. Elastin was imaged by its autofluorescence with a 525/50 filter. Fluorescence signals from smooth muscle cell nuclei were obtained with a 575/25 filter set. Collagen fibers were visualized by collecting second harmonic generation (SHG) signals from collagen molecules with a photomultiplier tube fitted with a 492SP filter set. A depth-series of images, each consisting of 512 × 512 pixels covering a 510 µm square region, was obtained at a rate of 0.5 fps with a 2 × averaging with an interval of 1 µm until the fibrous structure of elastin was undetectable. The specimen was then cleared in the clearing solution and imaged again in the same manner within the clearing solution. The images obtained were projected onto a radial-circumferential (*r *− *θ*) plane (standard deviation projection) to demonstrate the visibility depth in each condition.

A ring-shape specimen with an axial length of 1 mm was prepared from another isolated rat aorta by cutting in a plane transverse to the longitudinal axis of the aorta. Following the fluorescent labeling of smooth muscle cell nuclei in the specimen with 5 µM ethidium homodimer-III (Biotium) in PBS overnight at 4 °C, the specimen was imaged under the two-photon microscope (Nikon) in a non-cleared, normal state within PBS. Images on the radial-circumferential (*r *− *θ*) plane capturing all layers of elastin fibers, smooth muscle cells, and collagen fibers as described above. A depth-series of images, each consisting of 512 × 512 pixels covering a 347 µm square region, was obtained at a rate of 0.5 fps with a 2 × averaging with an interval of 0.5 µm until the signals became undetectable. The specimen was then cleared in the clearing solution and imaged again in a similar fashion within the clearing solution.

### Pressure-diameter test

To characterize the mechanical behavior of the aorta with/without tissue clearing, a pressure-diameter test was performed using three animals. Each end of the aorta specimen was attached to a custom-made stainless-steel hollow jig and connected to a testing device (Fig. 2a) similar to the ones used in previous studies^13,14^, with the abdominal side facing upward (Fig. 3). The length of the test section was at least 20 mm. The test was carried out at room temperature while the specimen was maintained in a bath of the device filled with PBS. The intraluminal pressure was provided using a hand pump and monitored with a mercury manometer (Navis, Japan). The images of the aorta were captured with a coupled-charged device (CCD) camera (DFC310, Leica, Germany) equipped with a stereomicroscope (M165 FC, Leica). The specimen was stretched to its in vivo length, and 10 cycles of inflation and deflation with PBS between 0 and 200 mmHg was applied as preconditioning. This was followed by a single cycle of inflation and deflation; the image of the aorta was captured at each pressure increment of 20 mmHg during the inflation and at each 20 mmHg reduction during the deflation for data analysis. The specimen was detached from the testing device and placed in the clearing solution for 2 h at room temperature until the aorta was completely cleared. The testing protocol was carried out again, with the specimen maintained in the clearing solution (Fig. 3). The series of tests were conducted on the same day of the collection of the aorta.

### Dynamic two-photon microscopy

Another three animals were used for dynamic two-photon microscopy. The aorta specimen was collected as described above and cell nuclei were stained with ethidium homodimer-III (Biotium) dissolved at 5 µM in PBS for 1 h at room temperature on a rocker. The specimen was then cleared in the clearing solution containing 5 µM ethidium homodimer-III overnight at 4 °C on a rocker. For microstructural observation of the aorta during intraluminal pressurization, the specimen was attached to the same testing device used for the pressure-diameter test mounted on a motorized stage of the two-photon microscope (Nikon), with the abdominal side facing upward and stretched to the in vivo length. The system was modified slightly for multiphoton microscopy (Fig. 2b). The aorta was kept in the bath filled with the clearing solution, and intraluminal pressure was applied as hydrostatic pressure by changing the height of a reservoir (25 mL syringe) filled with saturated saline solution. The saline in the reservoir and the clearing solution in the specimen were connected via a single air bubble introduced within the flow circuit. The pressure level was monitored with a pressure transducer (DX-100, Nihon Koden, Japan) located at the end of the specimen opposite to the end connecting to the reservoir.

Ten cycles of the inflation and the deflation of the aorta by the application of the intraluminal pressure between 0 and 160 mmHg were performed as preconditioning. This was followed by a serial application of 0, 40, 70, 100, and 130 mmHg to the specimen; microscopic images were captured at each pressure step as follows. To characterize alterations of the microscopic structure of the aorta during the pressurization, three major components of the aorta, namely, elastin, smooth muscle cells, and collagen were imaged simultaneously throughout the aortic wall, from the innermost intimal elastin layer to the outmost adventitial collagen layer. A depth-series consisting of a total of up to 200 images was captured with an interval of 0.67 or 1 µm; each image, a size of 512 × 512 pixels covering a 347 µm square region, was obtained at a rate of 0.5 fps with a 2 × averaging. The laser intensity and the sensitivity of the photomultiplier at each channel were fine-tuned to avoid halation.

### Alignment analysis

In the microscopic images obtained, alterations in the alignment of elastic fibers in each elastic lamina and smooth muscle cell nuclei in each smooth muscle layer were evaluated; two out of three specimens exhibited low collagen SHG intensity, and thus collagen fiber alignment analysis could only be performed with one specimen. To identify representative regions in each EL and SML at each pressure step, a 256 × 256 pixels square region was cropped from the entire depth-series images of EL and SML, which provided the most distinctive peaks and troughs in the profile of average signal intensity of each image slice along the depth-position (Fig. 5). The depth-position of each of 6 peaks for EL and 5 peaks for SML was identified, and the corresponding depth-slice was selected as a representative image of each of EL and SML and used for subsequent analysis.

The alignment of elastic fibers and smooth muscle cell nuclei was evaluated across the depth-series images as well as in the selected EL and SML images using Directionality function in ImageJ/Fiji (version 2.1.0, NIH, USA). This performed two-dimensional fast Fourier transform (2D-FFT), and the probability density function of the Gaussian distribution was fitted to the angular distribution of elastic fibers/nuclei in the images. From the analysis, three parameters describing their orientation were obtained: the peak (degree) and dispersion (unitless) of the fitted probability distribution function as well as the goodness of fit (a unitless value ranging between 0 and 1). Among the three parameters, we used the peak value as the representative alignment angle of elastic fibers/cell nuclei. In addition, changes in an overall, average peak alignment angle of ELs and SMLs in a whole aorta specimen were evaluated by simply calculating and comparing the mean and standard deviation of the peak and the dispersion of all the ELs and SMLs from two specimens at each pressure step.

### Strain analysis

To evaluate the deformation of each of EL, SML, and collagen fiber networks in EL and SML, an image-based strain analysis was performed. From the full-size depth-stack of each component at each pressure step, sub-stack images were collected covering the entire thickness of each layer; the depth-position of each layer was determined by visual inspection of *r *− *θ* (radial-circumferential) plane images reconstructed from the original depth-stack images of *z *− *θ* (axial-circumferential) plane. All images in each sub-stack were summed to create a projected image, and a total of 5 projected images (corresponding to 0, 40, 70, 100, and 130 mmHg) were stacked.

To set strain markers, characteristic objects in each image-stack, trackable throughout the stack series, were selected manually around the horizontal centerline of the images. In EL images, these characteristic features were bright spots in elastic fiber network; these were typical cell nuclei in SML. For circumferential strain, two markers positioned approximately the same horizontal locations across the horizontal centerline were paired. The distance between the paired markers was recorded at each pressure step, and the nominal strain for circumferential direction was calculated. At least three pairs of markers were used for strain measurement in each stack. Similarly, two markers positioned approximately the same vertical locations were paired and used for the determination of the axial strain.

### Statistical analysis

Statistical analyses were performed using the statistical language R (ver. 4.3.0). Comparisons among more than two groups were performed using Kruskal–Wallis test, followed by Steel–Dwass multiple comparison test if statistical significance was found in Kruskal–Wallis test. In the comparison of the alignment angle distribution, Dunnett test was used, taking the data of 0 mmHg as control. Significance in the difference between two independent correlations was assessed using R package *cocor*^40^ and Fisher’s *z* statistics were calculated. In all tests, the significance level was set at *P* < 0.05.

## Supplementary Information

Supplementary Figure 1.

Supplementary Figure 2.

Supplementary Figure 3.

Supplementary Figure 4.

Supplementary Figure 5.

Supplementary Figure 6.

Supplementary Figure 7.

## Data Availability

The datasets generated during and analyzed during the current study are available from the corresponding author on reasonable request.
