# Supplementary material for: Through the cleared aorta: three-dimensional characterization of mechanical behaviors of rat thoracic aorta under intraluminal pressurization using optical clearing method
Source: Sci Rep. 2022 May 23;12:8632. doi: 10.1038/s41598-022-12429-5 (PMC9126909; doi:10.1038/s41598-022-12429-5)
Supplement: Supplementary file 1 — Supplementary Figure 1. [file 41598_2022_12429_MOESM1_ESM.pdf]

Supplementary Figure S1

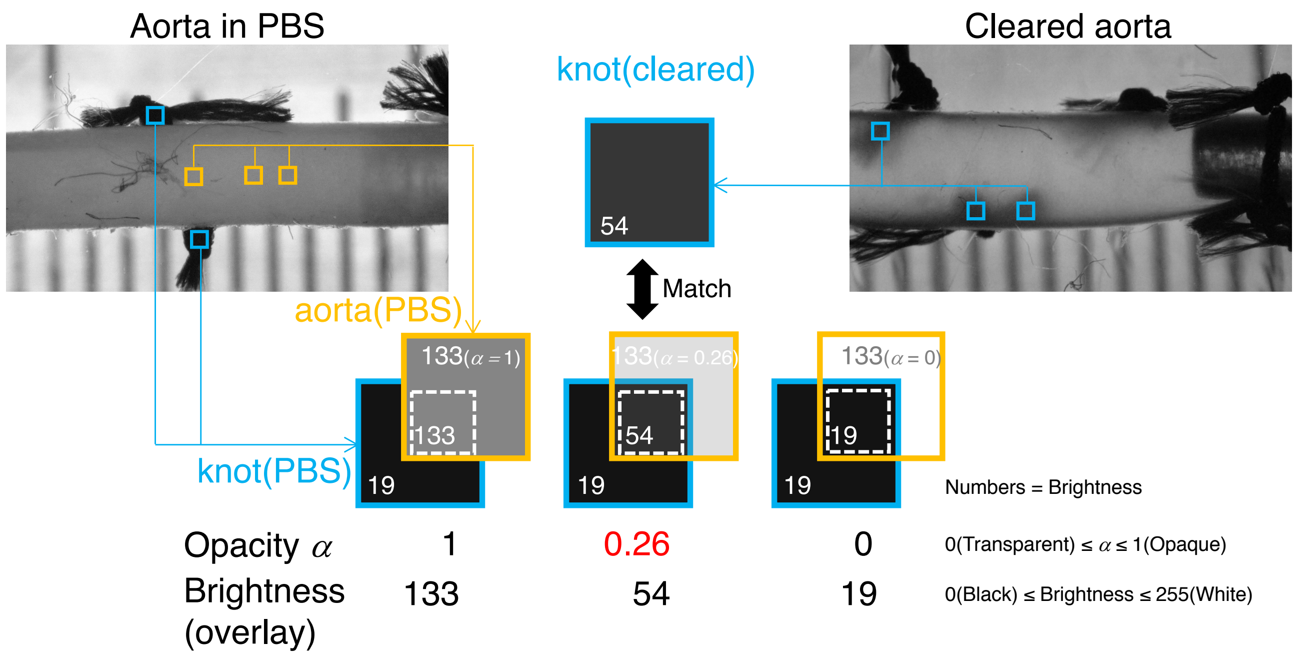

A representative example of the image analysis procedure for the determination of the opacity of the cleared aorta. To determine the opacity, two or three small regions of interest (ROI) were set on the image of the aorta before clearing, enclosing the aorta wall itself (yellow rectangles in the top left image). Another two to three small ROI were set on the same image of the aorta but enclosing the knots of the suture of closing intercostal arteries (which were very dark in the gray scale) (blue rectangles in the top left image). The same procedure was repeated for the image of the aorta after clearing, and the small ROI for the knots were set on those seen through the cleared aorta (which were not visible before clearing) (blue rectangles in the top right image). A gray scale brightness value (between 0 (black) and 255 (white) in a 8-bit image) was obtained from each ROI, and an average value was calculated for knot(PBS), knot(cleared), and aorta(PBS). A square image having the brightness of knot(PBS) (background layer) was overlayed by the same size of a square image having the brightness of aorta(PBS) (foreground layer). The opacity of the aorta(PBS), alpha value α, was defined as the opacity of the cleared aorta when the brightness of the overlay image (a small rectangle with white broken lines in the schematic) was matched to the brightness of knot(cleared);

$$\alpha \times g_1 + g_2 = g_{\mathrm{overlay}}$$

where g_1, g_2, and g_{overlay} is the brightness of aorta(PBS), knot(PBS), and the overlay image (= knot(cleared)), respectively, and the opacity α is between 0 (fully transparent) and 1 (fully opaque). This analysis was performed on the images from three experimental samples, and the resulting opacity of the cleared aorta was 0.18 ± 0.13 (mean ± SD, N = 3).
