# Supplementary material for: Through the cleared aorta: three-dimensional characterization of mechanical behaviors of rat thoracic aorta under intraluminal pressurization using optical clearing method
Source: Sci Rep. 2022 May 23;12:8632. doi: 10.1038/s41598-022-12429-5 (PMC9126909; doi:10.1038/s41598-022-12429-5)
Supplement: Supplementary file 2 — Supplementary Figure 2. [file 41598_2022_12429_MOESM2_ESM.pdf]

Supplementary Figure S2

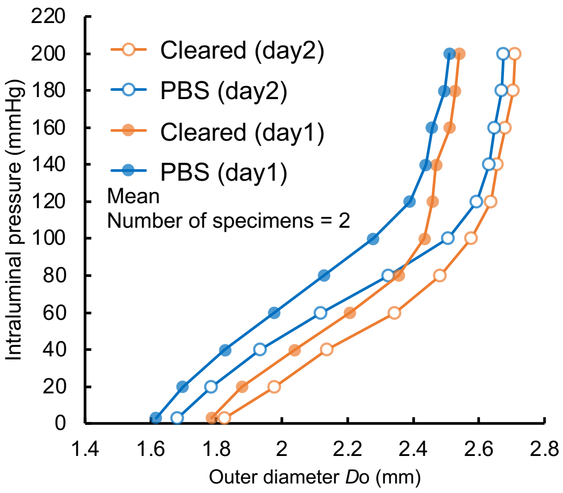

Pressure-diameter relationships of two rat thoracic aorta specimens tested in a normal state within PBS and in a cleared state within the clearing solution on day 2, along with the data obtained on day 1 (selected from the data in Figure 4).
