# Supplementary material for: Through the cleared aorta: three-dimensional characterization of mechanical behaviors of rat thoracic aorta under intraluminal pressurization using optical clearing method
Source: Sci Rep. 2022 May 23;12:8632. doi: 10.1038/s41598-022-12429-5 (PMC9126909; doi:10.1038/s41598-022-12429-5)
Supplement: Supplementary file 3 — Supplementary Figure 3. [file 41598_2022_12429_MOESM3_ESM.pdf]

Supplementary Figure S3

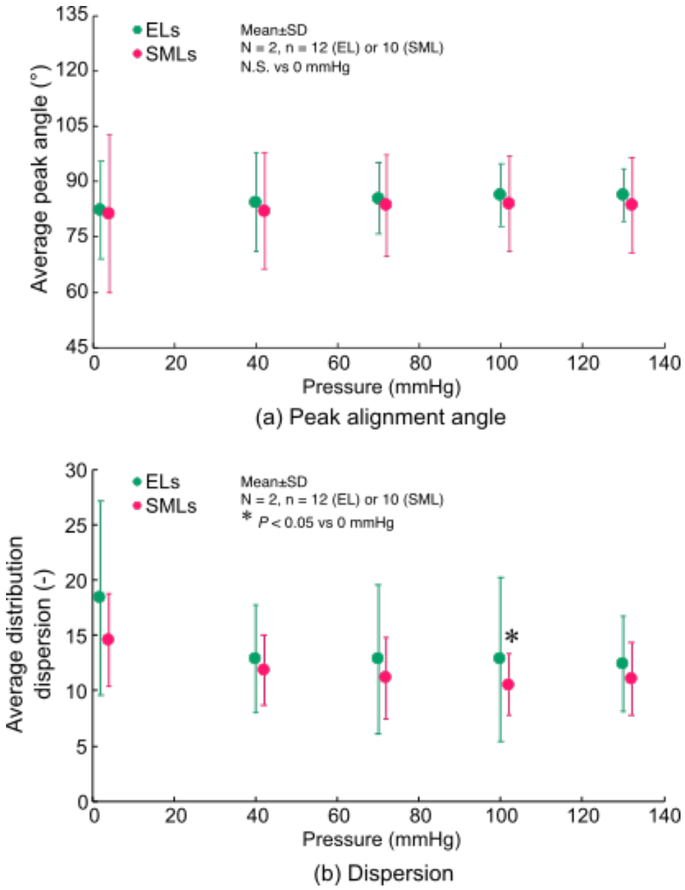


Changes in the overall, average alignment angle of elastic fibers in EL and SMC nuclei in SML in a whole aorta specimen during the pressurization. (**a**) Changes in the average peak angle; (**b**) Changes in the dispersion of the angle distribution. The peak angle at 90° corresponds to the circumferential direction of the aorta. These changes were not statistically significant, except that the dispersion of SMLs at 100 mmHg was significantly smaller than at 0 mmHg.
