# Supplementary material for: Through the cleared aorta: three-dimensional characterization of mechanical behaviors of rat thoracic aorta under intraluminal pressurization using optical clearing method
Source: Sci Rep. 2022 May 23;12:8632. doi: 10.1038/s41598-022-12429-5 (PMC9126909; doi:10.1038/s41598-022-12429-5)
Supplement: Supplementary file 4 — Supplementary Figure 4. [file 41598_2022_12429_MOESM4_ESM.pdf]

Supplementary Figure S4

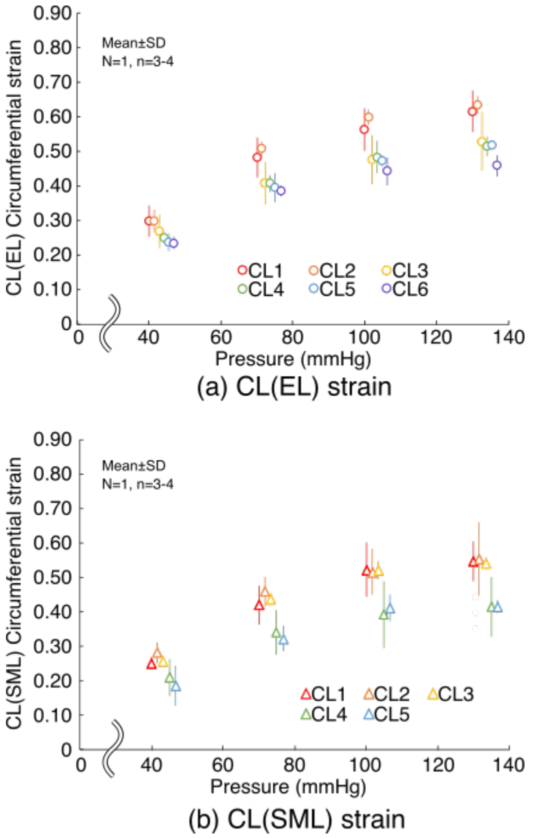

Circumferential strain in (**a**) collage fiber networks in ELs (CL(EL)) and (**b**) those in SMLs (CL(SML)), obtained from one sample, in which clear collagen SHG images were observed across the thickness of the aortic wall. N, number of specimens; n, the total number of the marker pairs analyzed.
