# Supplementary material for: Through the cleared aorta: three-dimensional characterization of mechanical behaviors of rat thoracic aorta under intraluminal pressurization using optical clearing method
Source: Sci Rep. 2022 May 23;12:8632. doi: 10.1038/s41598-022-12429-5 (PMC9126909; doi:10.1038/s41598-022-12429-5)
Supplement: Supplementary file 5 — Supplementary Figure 5. [file 41598_2022_12429_MOESM5_ESM.pdf]

Supplementary Figure S5

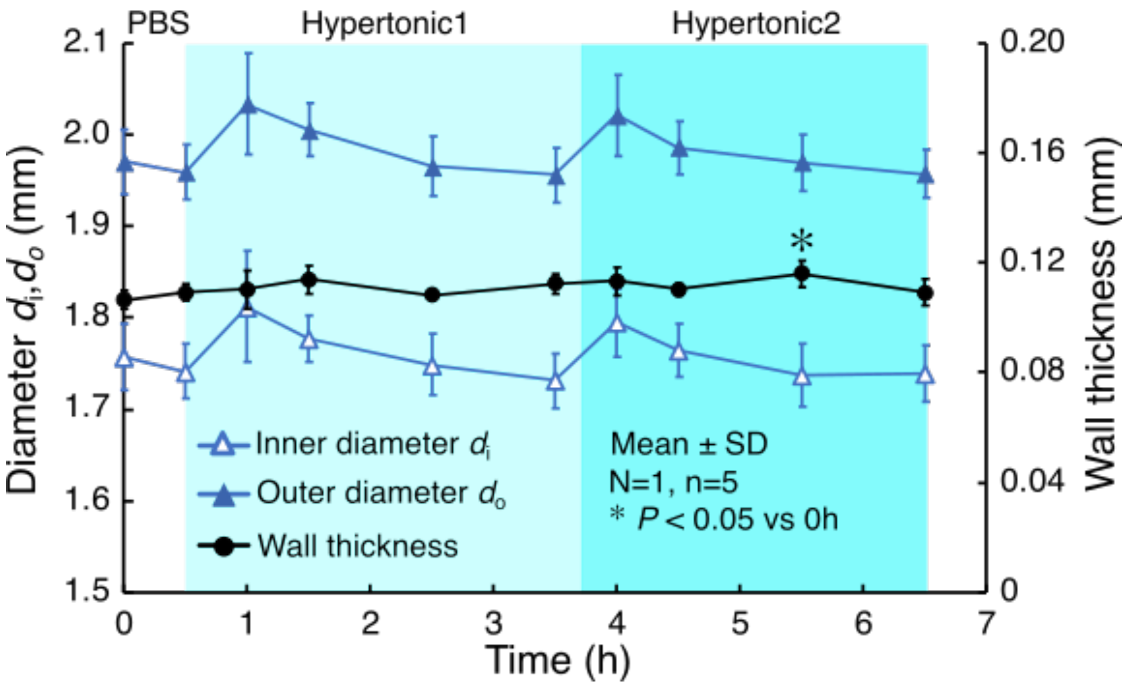

Temporal profile of the inner and outer diameter as well as the wall thickness of rat thoracic aorta incubated in ×1 PBS (PBS), ×1.25 hypertonic PBS (Hypertonic1), and ×1.5 hypertonic PBS (Hypertonic2). The inner and outer diameter of a ring-shape aorta specimen was measured in PBS. This was followed by incubation in hypertonic PBS (×1.25 higher concentration, approximately 372 mOsm/L) (Hypertonic1) for 3 h. The diameters were measured at 30 min, and every 60 min until 3 h using a stereomicroscopy. The specimen was further incubated in another hypertonic PBS with a higher osmolality (×1.5 higher concentration, approximately 447 mOsm/L) (Hypertonic2) for 3 h. Again, the diameters were measured at 30 min, and every 60 min until 3 h. It has been demonstrated that there was no statistically significant overall increase in the diameter of the aorta incubated within hypertonic PBS over the 6 h period. The wall thickness of the aorta exhibited a similar trend, only showing a significant increase at a single measurement time point.
