# Supplementary material for: Through the cleared aorta: three-dimensional characterization of mechanical behaviors of rat thoracic aorta under intraluminal pressurization using optical clearing method
Source: Sci Rep. 2022 May 23;12:8632. doi: 10.1038/s41598-022-12429-5 (PMC9126909; doi:10.1038/s41598-022-12429-5)
Supplement: Supplementary file 6 — Supplementary Figure 6. [file 41598_2022_12429_MOESM6_ESM.pdf]

Supplementary Figure S6

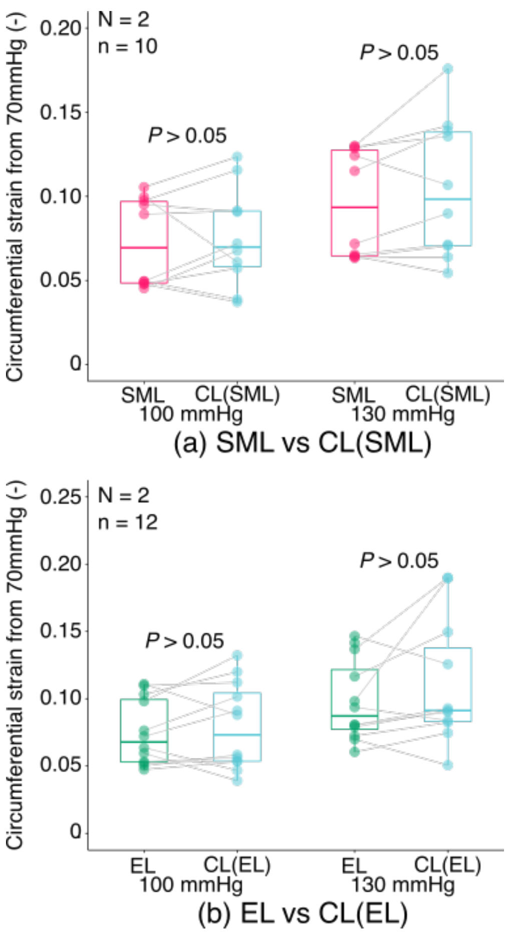


Comparisons in the magnitude of circumferential strain (**a**) between SML and CL(SML) and (**b**) between EL and CL(EL). Strain at an intraluminal pressure of 100 and 130 mmHg was calculated based on the distance between the strain markers at 70 mmHg from two aorta samples (one used in the analysis presented in Figure 8, and the other not used in Figure 8 due to a limited visibility of collagen SHG at low pressure levels). There were no statistically significant differences (assessed with paired-sample Wilcoxon rank-sum tests). N, number of specimens; n, the total number of the layers analyzed.
