# Supplementary material for: Through the cleared aorta: three-dimensional characterization of mechanical behaviors of rat thoracic aorta under intraluminal pressurization using optical clearing method
Source: Sci Rep. 2022 May 23;12:8632. doi: 10.1038/s41598-022-12429-5 (PMC9126909; doi:10.1038/s41598-022-12429-5)
Supplement: Supplementary file 7 — Supplementary Figure 7. [file 41598_2022_12429_MOESM7_ESM.pdf]

Supplementary Figure S7

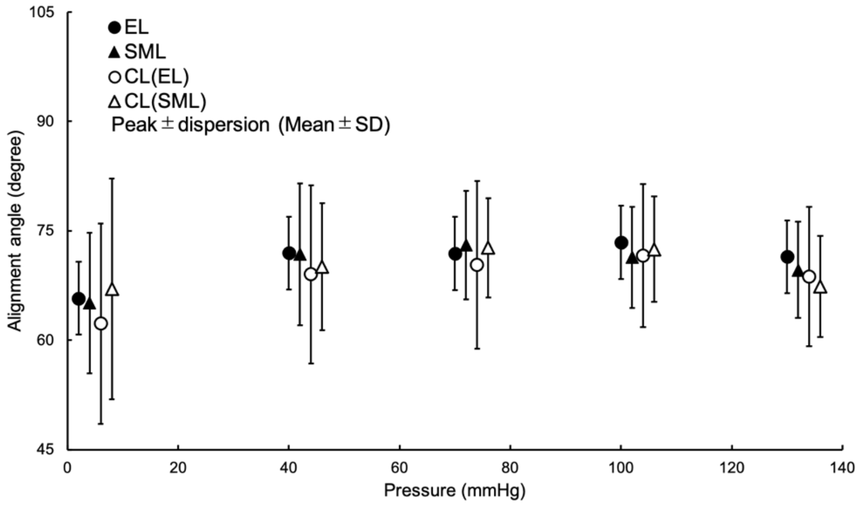

Representative example of alignment angle changes of elastin, smooth muscle cell nuclei, and collagen in EL and SML at 4th layer in one specimen through the pressurization. There were no marked differences among the components as well as between different pressure levels in each component.
